# Integrated Bone and Ligamentous Reconstruction of the Distal Radius After Oncologic Resection: Proximal Fibular Autograft Combined with Distal Oblique Bundle Reconstruction

**DOI:** 10.3390/life16030370

**Published:** 2026-02-25

**Authors:** Awad Dmour, Bogdan Puha, George Enescu, Adrian-Claudiu Carp, Bianca-Ana Dmour, Ștefan-Dragoș Tîrnovanu, Dragoș-Cristian Popescu, Liliana Savin, Norin Forna, Tudor Pinteala, Bogdan Veliceasa, Paul-Dan Sirbu

**Affiliations:** Grigore T. Popa University of Medicine and Pharmacy Iasi, 700020 Iasi, Romania; dmour-awad@umfiasi.ro (A.D.); adrian-claudiu.carp@umfiasi.ro (A.-C.C.); bianca-ana.dmour@umfiasi.ro (B.-A.D.); stefan-dragos.tirnovanu@umfiasi.ro (Ș.-D.T.); dragos.popescu@umfiasi.ro (D.-C.P.); liliana.savin@umfiasi.ro (L.S.); norin.forna@umfiasi.ro (N.F.); tudor_pinteala@umfiasi.ro (T.P.); bogdan.veliceasa@umfiasi.ro (B.V.); paul.sirbu@umfiasi.ro (P.-D.S.)

**Keywords:** giant cell tumor, distal radius, proximal fibular autograft, distal radioulnar joint, distal oblique bundle reconstruction

## Abstract

Campanacci grade III giant cell tumors of the distal radius frequently require en bloc resection to achieve adequate oncologic control. Reconstruction of the resulting defect remains challenging, particularly with respect to preservation of distal radioulnar joint stability and forearm rotation. Although proximal fibular autograft reconstruction is well established, ligamentous stabilization of the distal radioulnar joint is rarely incorporated in oncologic settings. This technical note describes an integrated reconstructive strategy combining proximal fibular autograft with distal oblique bundle reconstruction, illustrated by a representative clinical case. The technique involves segmental en bloc resection of the distal radius followed by reconstruction using an ipsilateral, nonvascularized proximal fibular autograft including the fibular head. Distal radioulnar joint stability is addressed through reconstruction of the distal oblique bundle using an autologous palmaris longus tendon graft. Surgical indications, operative steps, donor site stabilization, and perioperative management are detailed. Functional evolution was assessed using the Musculoskeletal Tumor Society scoring system and range-of-motion measurements. Histopathological examination confirmed negative oncologic margins. Early postoperative events included donor-site common peroneal nerve dysfunction and radiocarpal instability requiring temporary Kirschner wire stabilization. At nine months, the Musculoskeletal Tumor Society score reached 80%, with forearm rotation preserved at 68.8% pronation and 81.3% supination of normal values. Combined osseous and ligamentous reconstruction following distal radius resection is technically feasible and may allow preservation of distal forearm mechanics while maintaining oncologic principles. Broader validation will require application in larger clinical series and longer follow-up.

## 1. Introduction

Giant cell tumors (GCT) are primary bone tumors that are histologically benign but may exhibit aggressive local behavior, conferring significant clinical relevance in orthopedic oncology. They are composed of mononuclear stromal cells associated with multinucleated osteoclast-like giant cells and are characterized by pronounced osteolytic activity. In selected cases, denosumab has been used as adjunctive therapy to reduce tumor activity. Surgical resection remains the definitive treatment for aggressive lesions [1,2]. Although GCT account for approximately 4 to 5 percent of all primary bone tumors, their frequent involvement of the epiphyseal regions of long bones, particularly in proximity to major joints, presents substantial therapeutic challenges [3,4].

GCTs most commonly occur during the third and fourth decades of life and show a higher prevalence among young women. They predominantly affect the appendicular skeleton, with a notable predilection for the distal epiphysis of the radius [5]. This location represents approximately 10 to 12 percent of all reported GCT cases [6,7]. Due to the close anatomical relationship with both the radiocarpal joint and the distal radioulnar joint (DRUJ), tumors arising at this site have a direct impact on forearm biomechanics. Consequently, surgical management must balance oncologic radicality with strategies aimed at preserving wrist motion and forearm rotation [8,9,10].

Radiological staging plays a central role in guiding therapeutic decision making. The Campanacci classification, widely adopted in clinical practice and summarized in Table 1, categorizes giant cell tumors into three grades based on radiographic appearance and local behavior [11].

While this classification provides a useful framework for treatment planning, it does not always fully reflect biological behavior. Although traditionally considered benign, Campanacci grade III giant cell tumors of the distal radius demonstrate a reported pulmonary metastasis rate of approximately 2 to 5 percent, an atypical feature for benign bone lesions [4,12]. This behavior has been associated with increased mitotic activity and, in some cases, repeated surgical intervention, underscoring the need for a radical and carefully planned oncologic approach.

Beyond oncologic considerations, distal radius GCTs pose a significant challenge in achieving durable local control. Owing to the complex anatomy of the distal forearm and its proximity to functionally critical joints, extended curettage, despite being less invasive and potentially associated with superior short-term function, has been linked to high recurrence rates, reported in some series to reach up to 88 percent [7,8,13,14]. For lesions classified as Campanacci grade II with marked cortical thinning and for grade III tumors, segmental en bloc resection followed by reconstruction of osseous continuity and joint stability is therefore generally recommended [15].

Reconstruction following distal radius resection may be achieved using several strategies, including vascularized or nonvascularized fibular autografts, osteoarticular allografts, wrist arthrodesis, or customized prosthetic implants. Nonvascularized fibular autografts are frequently employed, particularly in settings with limited resources, due to their immediate availability, absence of immunogenic response, and favorable biological integration [8,13]. However, while these techniques can restore distal radial morphology and allow preservation of acceptable wrist function, they do not consistently address residual biomechanical instability, particularly at the level of the distal radioulnar joint [16].

Persistent DRUJ instability following distal radius reconstruction may manifest clinically as pain, restricted forearm rotation, or recurrent subluxation. To address this limitation, complementary ligamentous reconstruction techniques have been proposed. Reconstruction of the distal oblique bundle (DOB), a key stabilizing component of the distal interosseous membrane responsible for controlling dorsal and volar translation of the ulna relative to the radius, has emerged as a promising option [17]. Biomechanical studies have demonstrated the critical role of the DOB in maintaining DRUJ stability and facilitating pronation and supination [18,19]. Reconstruction using an autologous palmaris longus tendon graft, accessible through a volar approach, has been shown to provide biomechanical stability comparable to established techniques such as the Adams procedure [20].

To our knowledge, distal oblique bundle reconstruction has been described primarily in the treatment of distal radioulnar joint instability in nononcologic settings, while its systematic integration as an adjunct to biological distal radius reconstruction after tumor resection has been rarely reported [21,22]. Despite its recognized biomechanical importance in stabilizing the distal radioulnar joint, this ligamentous structure has received limited attention in oncologic distal radius reconstruction. In the absence of restoration of the distal oblique bundle, even anatomically accurate bony reconstruction may remain functionally compromised, particularly in young and active individuals.

In light of these considerations, contemporary management of distal radius giant cell tumors should extend beyond restoration of bone continuity and aim for integrated functional reconstruction. Surgical strategies that combine oncological resection with targeted ligamentous stabilization may improve distal radioulnar joint stability and forearm function.

**Figure 1:**
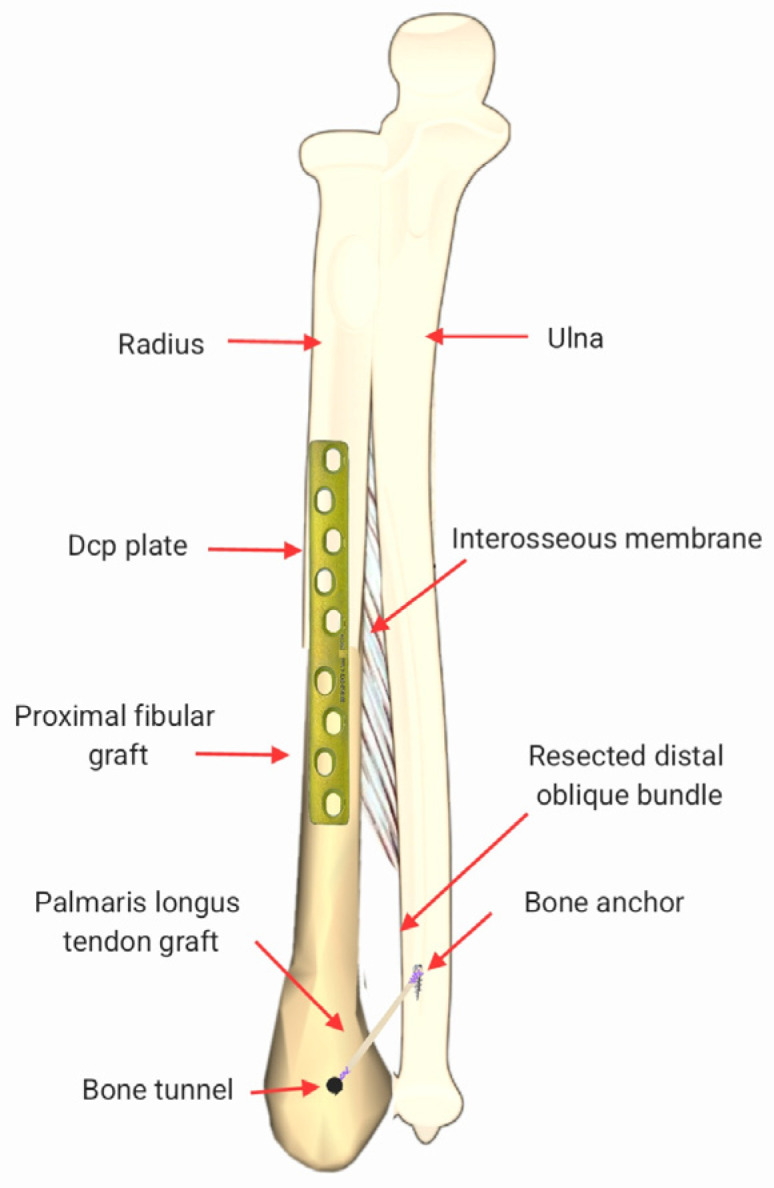
Schematic illustration of complex distal forearm reconstruction using a proximal fibular bone autograft combined with distal oblique bundle (DOB) reconstruction, performed with an autologous palmaris longus tendon graft fixed to the ulna using a metallic anchor.

This manuscript presents a technical note describing an integrated osseous and ligamentous reconstructive strategy following oncologic distal radius resection, illustrated by a representative clinical case (Figure 1).

## 2. Materials and Methods

### 2.1. Study Design

This manuscript is presented as a technical note describing an integrated surgical reconstruction strategy, illustrated by a representative clinical case. The purpose of the clinical data is to demonstrate feasibility, safety, and short-term functional evolution of the technique rather than to perform outcome analysis (Figure 1).

### 2.2. Surgical Indications and Contraindications

Indications

• Young or active patient with high functional demand;
• Requirement to preserve forearm rotation and wrist mobility;
• Absence of advanced radiocarpal or distal radioulnar joint degeneration.

Contraindications

• Extensive carpal involvement precluding autologous reconstruction;
• Severe preexisting distal radioulnar joint arthritis;
• Inability to comply with prolonged immobilization and rehabilitation.

### 2.3. Surgical Technique

#### 2.3.1. Preoperative Imaging Assessment

**Figure 2:**
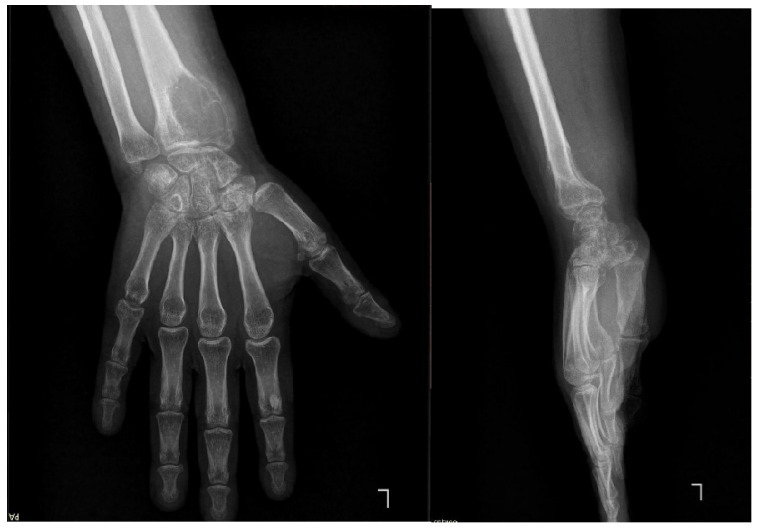
Anteroposterior and lateral wrist radiographs demonstrating an expansile osteolytic lesion at the level of the distal epiphysis of the left radius, suggestive of a giant cell tumor (GCT).

Preoperative imaging evaluation was performed using standard radiography and magnetic resonance imaging. Plain radiographs demonstrated an expansile osteolytic lesion involving the distal radius with cortical destruction (Figure 2). Magnetic resonance imaging was used to assess intraosseous extension, cortical breach, and involvement of adjacent soft tissues, providing essential information for surgical planning and determination of resection margins. Imaging findings were consistent with an aggressive distal radius lesion corresponding to Campanacci grade III.

#### 2.3.2. Preoperative Biopsy and Histopathological Confirmation

Preoperative tissue diagnosis was established prior to definitive surgical treatment. An incisional biopsy was performed through a volar approach, deliberately planned along the anticipated definitive surgical incision in order to avoid contamination of uninvolved compartments. The biopsy tract was positioned to allow subsequent en bloc excision during tumor resection. Histopathological examination confirmed the diagnosis of giant cell tumor of bone, characterized by a proliferation of mononuclear stromal cells associated with multinucleated osteoclast-like giant cells. Based on combined radiological and histopathological findings, the lesion was classified as Campanacci grade III.

#### 2.3.3. Resection Principles and Surgical Approach

As the initial operative step, the anterior biopsy tract was excised en bloc together with its surrounding soft tissue margins to minimize the risk of local tumor dissemination. The procedure was performed under general anesthesia with the patient in the supine position. A modified Henry approach was utilized to access the volar aspect of the distal forearm. This approach was chosen to allow safe oncologic exposure while respecting anatomical planes and facilitating subsequent reconstruction. Dissection was carried out through the interval between the brachioradialis and the flexor carpi radialis, with careful identification and protection of the radial artery. Deeper dissection proceeded between the flexor pollicis longus and the pronator quadratus, which was elevated to expose the volar surface of the distal radius. The median nerve was identified early and protected throughout the procedure.

**Figure 3:**
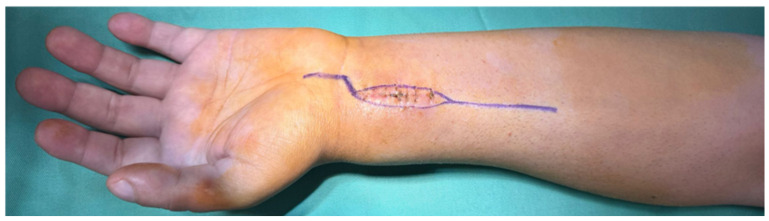
Extended modified Henry approach with excision of the anterior biopsy tract and midline extension for concomitant carpal tunnel release.

Extension of the volar approach toward the midline allowed concomitant release of the transverse carpal ligament, providing decompression of the carpal tunnel and reducing the risk of postoperative median nerve compression following extensive volar dissection and reconstruction (Figure 3).

**Figure 4:**
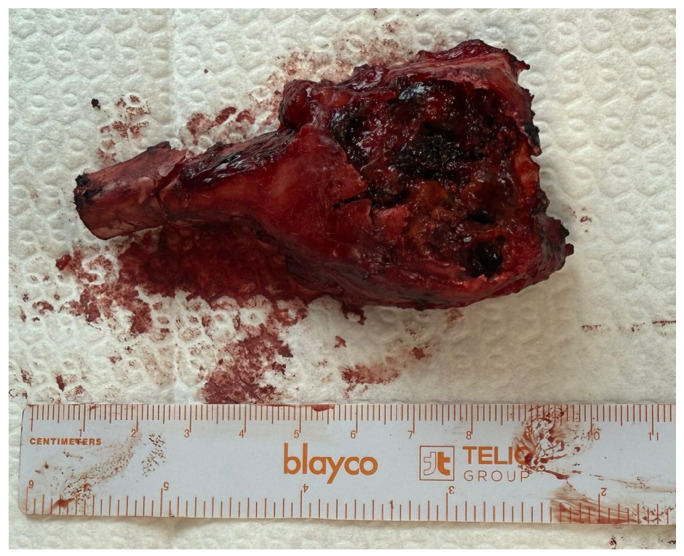
En bloc resected distal radius specimen demonstrating aggressive tumor morphology with destruction of the dorsal cortex.

Following complete exposure, en bloc resection of the distal radial segment was performed, including all involved soft tissue components and the joint capsule, while respecting oncological safe margins (Figure 4).

#### 2.3.4. Fibular Graft Harvest and Preparation

**Figure 5:**
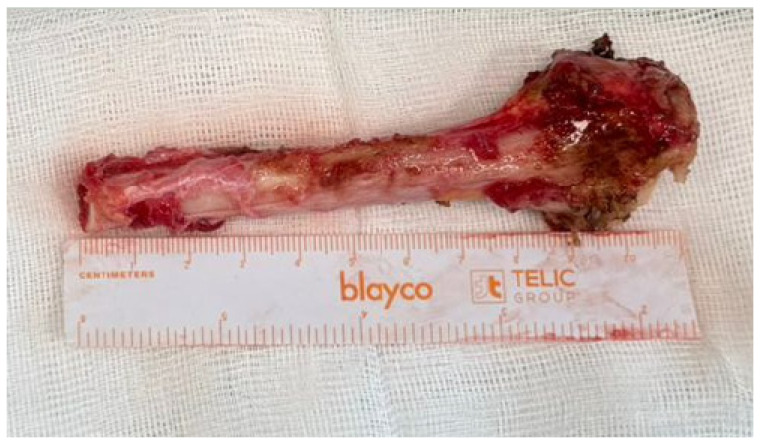
Proximal fibular head graft used for reconstruction of the resected distal radius segment, measured intraoperatively.

Following completion of distal radius resection, the length of the excised radial segment was measured intraoperatively. To compensate for potential graft settling and to ensure appropriate restoration of forearm length, a proximal fibular graft approximately 1 cm longer than the resected segment was harvested (Figure 5).

**Figure 6:**
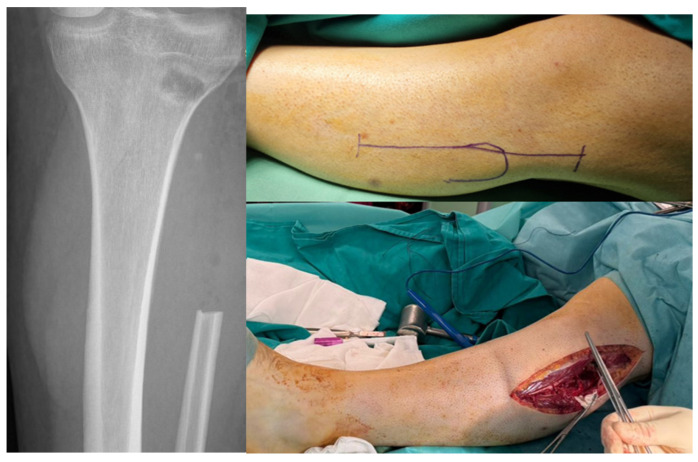
Radiographic and intraoperative images illustrating the proximal fibular head harvest site, with identification and isolation of the common peroneal nerve, and restoration of knee stability through reinsertion of the biceps femoris tendon and the lateral collateral ligament onto the proximal tibial epiphysis using a resorbable anchor.

The graft was obtained from the ipsilateral proximal fibula, including the fibular head, using a standard lateral approach. During exposure and graft harvest, the common peroneal nerve was carefully identified, dissected free, and protected throughout the procedure. Care was taken to preserve surrounding soft tissue attachments intended for subsequent ligamentous reinsertion. After harvest, the graft was prepared on the instrumentation table and contoured to reproduce the anatomical characteristics of the resected distal radius. Particular attention was paid to graft orientation, ensuring that the curvature and articular geometry of the fibular head were aligned to approximate the native distal radial anatomy and maintain physiological ulnar variance relative to the contralateral limb (Figure 6).

#### 2.3.5. Donor Site Stabilization and Lateral Collateral Ligament Reconstruction

Following harvest of the proximal fibular graft, reconstruction of the lateral stabilizing structures of the knee was performed to preserve donor site stability. The lateral collateral ligament and the biceps femoris tendon were reinserted onto the proximal tibial epiphysis using a resorbable PushLock^®^ PEEK anchor (Arthrex, Inc., Naples, FL, USA). Reinsertion was performed with the knee positioned in slight flexion in order to restore physiological ligament tension and lateral knee stability.

Care was taken to reproduce the native anatomical orientation of the lateral collateral ligament complex. Intraoperative assessment confirmed adequate lateral knee stability following reinsertion. Additional cancellous bone graft harvested from the proximal tibia was used to supplement the donor site as needed. Postoperatively, the knee was protected with an orthosis to limit varus stress during the early healing phase.

#### 2.3.6. Fixation Technique and Graft Positioning

**Figure 7:**
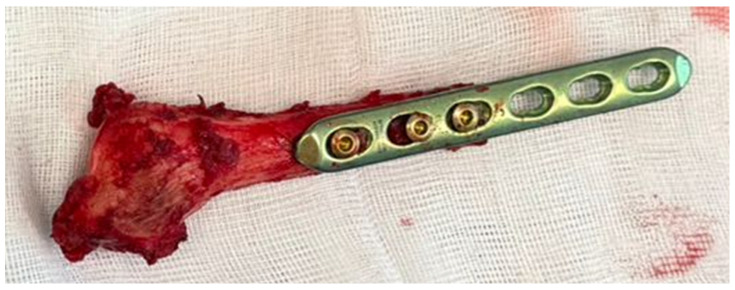
Proximal fibular graft prepared with plate fixation prior to definitive internal fixation.

Fixation was initiated by securing the plate to the fibular graft on the instrumentation table prior to definitive implantation. This step allowed accurate control of graft length, rotational alignment, and overall geometry before attachment to the native bone. Preassembly of the plate–graft construct facilitated precise positioning and minimized intraoperative adjustment following implantation (Figure 7).

**Figure 8:**
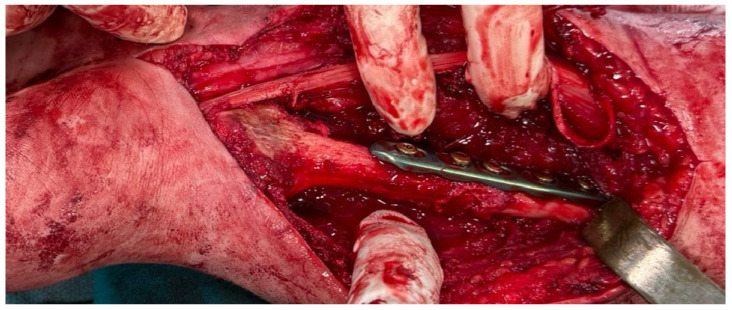
Intraoperative image showing osteosynthesis of the proximal fibular graft to the remaining radial diaphysis.

The assembled construct was subsequently fixed to the remaining radial diaphysis, restoring forearm length and axial alignment while respecting the anatomical orientation of the fibular graft relative to the native radius. Internal fixation was achieved using a locking compression plate, providing stable fixation of the reconstruction and allowing early structural stability (Figure 8).

#### 2.3.7. Distal Oblique Bundle Reconstruction

**Figure 9:**
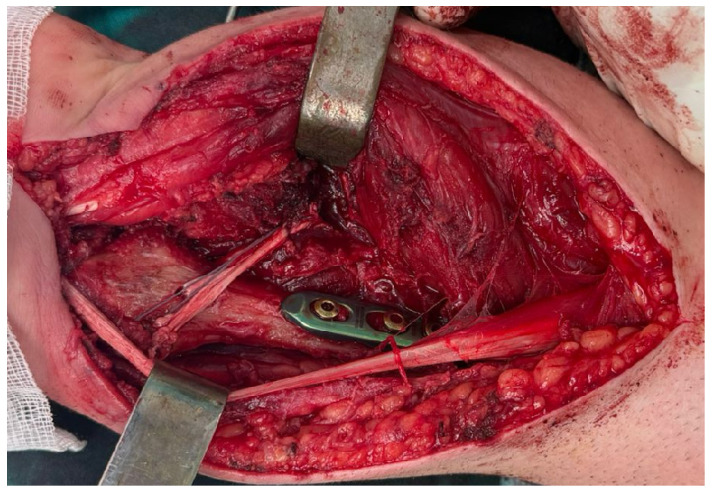
Intraoperative image showing the final reconstructive construct, including the proximal fibular graft and distal oblique bundle (DOB) reconstruction using bone tunnels and a bone anchor.

To address distal radioulnar joint stability, reconstruction of the distal oblique bundle was performed using an autologous tendon graft harvested from the palmaris longus. For graft passage, a trans-osseous tunnel was created in the fibular graft in an oblique direction oriented toward the ulnar insertion site, replicating the native course of the distal interosseous membrane toward the distal ulna. The tendon graft was tensioned with the forearm positioned in neutral rotation to reproduce physiological restraint while avoiding over-constraint of distal radioulnar motion. Fixation to the distal ulna was performed using a metallic anchor (Corkscrew^®^, Arthrex, Inc., Naples, FL, USA) placed at the planned ulnar insertion, and stability was verified clinically by assessing dorso-volar translation under gentle stress, with the objective of restoring physiological ligamentous tension (Figure 9) [22,23].

Residual dorsal and lateral capsular structures were sutured to the fibular graft through transosseous tunnels in order to enhance soft tissue stabilization. To protect both the osseous and the DOB reconstruction during the early healing phase, a temporary Kirschner wire between the fibular graft and ulna was placed.

#### 2.3.8. Intraoperative Assessment and Postoperative Management

**Figure 10:**
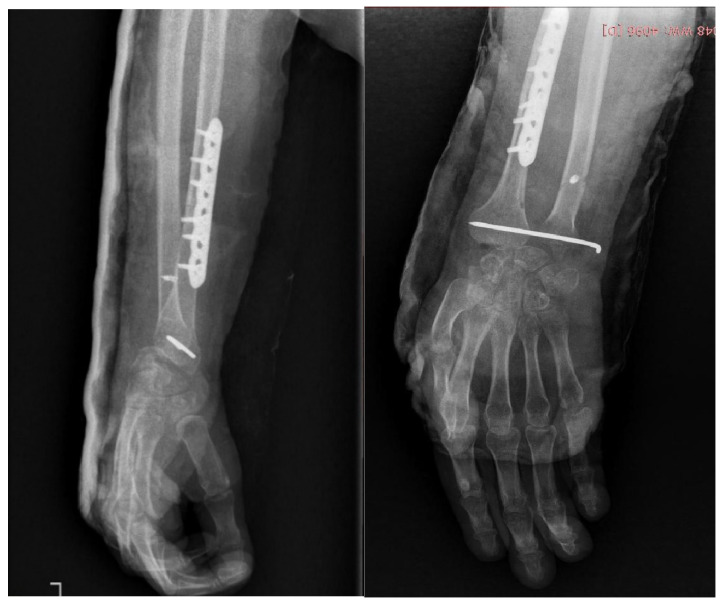
Postoperative control radiographs (anteroposterior and lateral views) of the wrist joint. The lateral view demonstrates early postoperative dorsal subluxation of the carpal bones, while the anteroposterior view shows correct positioning of the proximal fibular graft and plate osteosynthesis with K wire in its correct place.

**Figure 11:**
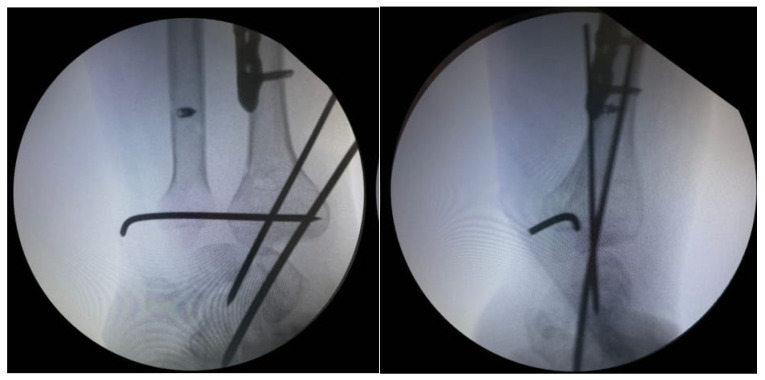
Intraoperative fluoroscopic images demonstrating Kirschner wire fixation for stabilization of the carpal bones and restoration of radiocarpal joint congruence.

Intraoperative radiographic control confirmed appropriate positioning and alignment of the reconstructive components. Early postoperative radiographic evaluation demonstrated dorsal subluxation of the carpal bones (Figure 10), attributed to residual ligamentous instability and the absence of distal cortical support. Surgical reintervention was performed consisting of temporary carpal stabilization using two transverse Kirschner wires, resulting in restoration of radiocarpal alignment (Figure 11).

Temporary radioulnar stabilization using a Kirschner wire was used as a protective measure to reduce early micromotion and to support healing of the reconstructed soft tissue stabilizers in the setting of a complex reconstruction. This step was selected to minimize the risk of early instability during the immobilization phase, particularly when residual laxity may persist despite reconstruction and capsular repair.

The resected specimen was submitted for definitive histopathological examination to confirm the diagnosis and evaluate surgical margins. Postoperatively, the upper limb was immobilized in a long arm cast to limit pronation and supination and to protect the ligamentous reconstruction and graft integration. Immobilization was maintained for eight weeks, followed by initiation of a standardized rehabilitation protocol focused on gradual restoration of wrist motion and rebalancing of radioulnar rotation.

#### 2.3.9. Technical Pearls and Pitfalls

• Careful identification and protection of the common peroneal nerve during graft harvest is essential to minimize traction-related neuropraxia.
• Accurate intraoperative measurement of the resected radial segment is critical. Even small discrepancies in graft length may significantly alter wrist biomechanics. An excessively long graft may increase radiocarpal compression and dorsal carpal translation, whereas an undersized graft may result in positive ulnar variance and ulnar-sided wrist pain.
• Correct rotational alignment of the fibular head is important to approximate the curvature of the native distal radius and to optimize carpal articulation.
• Restoration of neutral ulnar variance prior to distal oblique bundle fixation is critical to avoid over-tensioning.
• In this technique, the tendon graft was fixed to the ulna using a suture anchor rather than a transosseous tunnel.
• An anteroposterior bone tunnel was created in the fibular graft just distal to where the sigmoid notch should have been, allowing the graft to pass obliquely toward the ulnar anchor.
• The tunnel position relative to the reconstructed articular surface is critical (placement too proximal may compromise articular support, whereas placement too distal may reduce the stabilizing vector of the distal oblique bundle).
• Anchor fixation on the ulna should be positioned slightly proximal to the level of the graft tunnel to reproduce the native oblique orientation of the distal interosseous membrane.
• The tendon graft should be tensioned with the forearm in neutral rotation to prevent rotational over-constraint.
• Temporary radioulnar stabilization may be considered in cases with residual laxity after reconstruction, particularly when soft tissue tension remains uncertain (for example when dorsal capsule resection was performed)
• Early radiographic surveillance is recommended to detect subtle radiocarpal instability and to allow prompt intervention if alignment changes occur.
• Donor-site knee stability must be restored through appropriate reinsertion of the lateral collateral ligament and biceps femoris tendon with adequate tension and secure anchor fixation, followed by protected postoperative immobilization.

## 3. Results

### 3.1. Early Postoperative Course

The patient underwent en bloc distal radius resection followed by reconstruction with a proximal fibular autograft and distal oblique bundle stabilization. Within the first 24 h postoperatively, partial motor deficit of the common peroneal nerve was identified at the donor site, without associated sensory disturbance. The presentation was consistent with traction-related neuropraxia. Conservative management with supervised physiotherapy was instituted. At nine months, progressive motor recovery was observed, although full neurological restitution had not yet been achieved. The residual deficit did not produce clinically relevant gait instability or limitation in daily activities.

**Figure 12:**
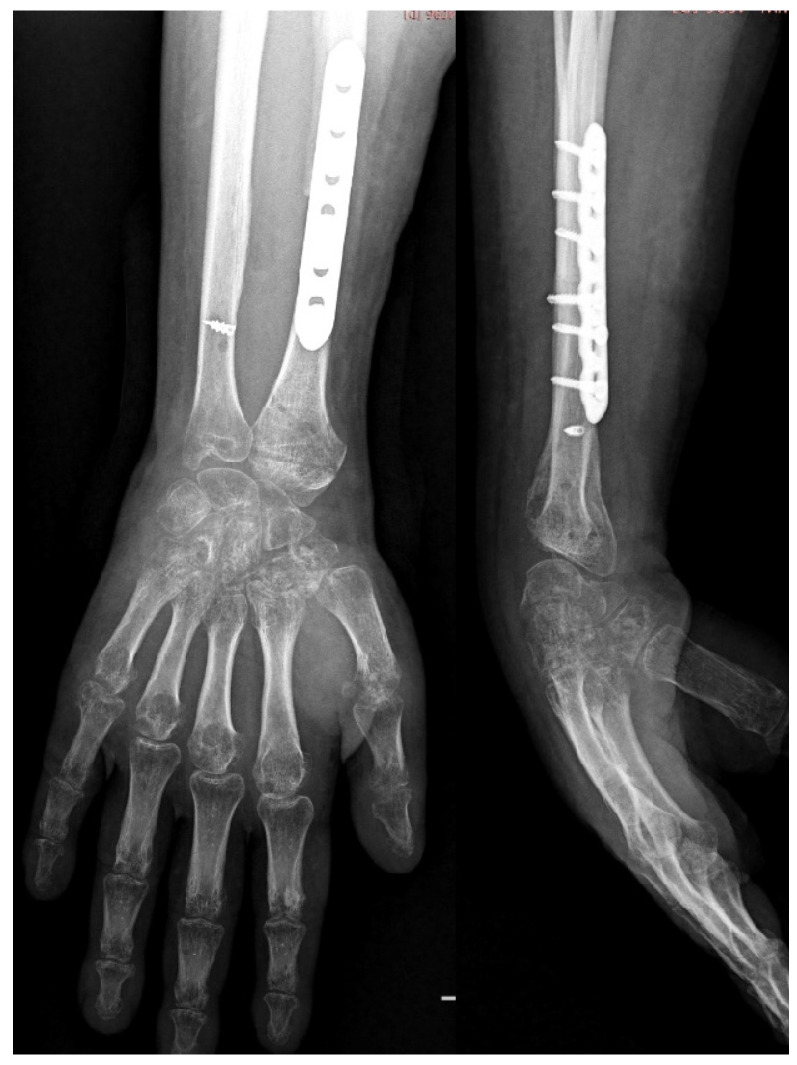
Three-month postoperative control radiographs (anteroposterior and lateral views) of the wrist joint, obtained after removal of the Kirschner wires.

Early postoperative radiographic evaluation identified dorsal subluxation of the carpal bones, reflecting residual radiocarpal instability. Surgical reintervention was undertaken, consisting of temporary stabilization of the carpus using two transverse Kirschner wires. Subsequent imaging confirmed restoration of radiocarpal alignment and congruence of the reconstruction (Figure 12). Histopathological examination confirmed Campanacci grade III giant cell tumor with negative resection margins.

### 3.2. Functional Evolution

Functional outcome was assessed using the Musculoskeletal Tumor Society (MSTS) scoring system (Table 2) and wrist range-of-motion measurements (Table 3). The MSTS score was 30.0% at one month, 33.3% at three months, and 80.0% at nine months postoperatively.

At nine months, forearm rotation was relatively preserved, with pronation and supination reaching 68.8 percent and 81.3 percent of normal values, respectively, whereas wrist flexion remained limited compared to contralateral values, The observed forearm rotation values are consistent with previously reported outcomes after proximal fibular reconstruction [2,13].

## 4. Discussion

Distal oblique bundle reconstruction is an established concept for stabilization of the distal radioulnar joint, particularly in the setting of nononcologic instability; however, its application in oncologic distal radius reconstruction remains limited in the available literature [21,22,24]. In contrast, segmental reconstruction of the distal radius using a proximal fibular autograft represents a well-recognized treatment option for giant cell tumors classified as Campanacci grade III, especially in cases in which preservation of the radiocarpal joint is not feasible [15,25]. This approach remains one of the most commonly employed reconstructive strategies, allowing restoration of forearm alignment and structural continuity while avoiding the increased technical complexity and morbidity associated with vascularized grafts or custom prosthetic implants [9]. Prosthetic reconstruction of the distal radius represents another available option, particularly in elderly patients or in cases with extensive joint destruction, although concerns regarding implant longevity, loosening, and limited restoration of forearm rotation have restricted its widespread use in young and active individuals [26,27].

Despite its widespread use, reconstruction with a proximal fibular graft does not fully restore normal wrist biomechanics and has been associated with a substantial incidence of postoperative carpal instability and progressive degenerative changes. Previous studies have reported postoperative subluxation and the development of wrist arthrosis in up to 50 percent of cases over time [8,16]. Nevertheless, a recent systematic review documented a patient satisfaction rate of 87 percent following proximal fibular reconstruction, suggesting that these functional limitations are often clinically acceptable, particularly in young and active patients [16]. Early postoperative instability, when promptly recognized and addressed, does not necessarily compromise subsequent functional recovery, emphasizing the importance of close radiographic and clinical surveillance during the immediate postoperative period.

An important aspect of the reconstructive strategy discussed in this study is the integration of distal oblique bundle reconstruction, a ligamentous structure that plays a key role in stabilizing the distal radioulnar joint during forearm rotation but is rarely addressed in oncologic distal radius reconstruction [18,24]. Reconstruction using an autologous palmaris longus tendon graft was intended to restore distal radioulnar stability and reduce the risk of secondary instability following segmental bone replacement. Biomechanical investigations have demonstrated that distal oblique bundle reconstruction can achieve stability comparable to the native ligament, with only minimal differences reported in isolated analyses of forearm supination [20].

Stability of the distal radioulnar joint depends on the combined contribution of bony congruence and surrounding soft tissue stabilizers, both of which are critical determinants of forearm rotation and overall functional outcome. Recent literature underscores the importance of preserving or reconstructing these stabilizing elements when interpreting postoperative joint stability after complex distal radius reconstruction procedures [28,29,30]. Compared with more extensive ligamentous reconstructions such as the Adams procedure, distal oblique bundle reconstruction requires a single surgical approach and a shorter tendon graft, potentially reducing operative time and surgical morbidity [31]. Moreover, current evidence suggests that isolated reconstruction of the distal oblique bundle is sufficient to restore functional stability in selected cases, without clear additional benefit from more extensive multi-ligamentous procedures [21].

Preservation of articular cartilage constitutes another relevant consideration in the oncologic management of distal radius tumors. Retention of the cartilage surface may act as a biological barrier against local tumor recurrence, whereas wrist arthrodesis necessitates cartilage removal and may increase vulnerability of the carpal bones to recurrence while limiting reconstructive options in the event of local relapse [8,14,32]. From this perspective, autologous reconstruction strategies that preserve joint structures may offer advantages in selected patients.

The combined use of proximal fibular autograft and DOB reconstruction appears to represent an effective approach for restoring distal forearm stability following oncologic resection, particularly in young patients for whom preservation of joint forearm rotation and functional independence is essential. Available evidence suggests that early postoperative instability, when adequately managed, does not preclude satisfactory medium-term functional recovery [32,33]. The absence of local recurrence and the lack of requirement for secondary wrist arthrodesis further support the oncologic safety and functional reliability of this reconstructive strategy [4,6,9,32].

Donor site morbidity remains an important consideration following proximal fibular head harvest. Transient common peroneal nerve dysfunction represents a recognized complication, with reported incidence rates ranging from 2 to 27 percent, although most cases demonstrate spontaneous recovery under conservative management and structured physiotherapy [34]. The transient neuropraxia observed in this case is consistent with previously reported incidence rates following proximal fibular harvest and demonstrated gradual recovery under conservative management. Preservation of knee stability is equally critical, and reinsertion of the biceps femoris tendon and lateral collateral ligament onto the proximal tibia using suture anchors has been shown to restore lateral stability with biomechanical properties comparable to native insertions [35]. Clinical series have reported low rates of clinically significant postoperative knee instability following this reconstructive approach [36,37].

Functional assessment using the Musculoskeletal Tumor Society score demonstrated modest early postoperative values, followed by gradual improvement during the initial follow-up period, particularly in pain control and emotional acceptance. This pattern likely reflects early adaptation to altered forearm biomechanics following reconstruction. Functional recovery in this context should be interpreted beyond isolated range-of-motion measurements and considered in relation to the patient’s ability to resume daily, professional, and recreational activities [4,6,9,32]. While the MSTS score provides a standardized and widely accepted tool for evaluating functional outcomes after oncologic upper limb reconstruction, its limitations in capturing wrist-specific and fine rotational deficits should be acknowledged. Further studies, ideally multicentric and comparative in design, are required to better define long term functional outcomes and to validate the broader applicability of combined osseous and ligamentous reconstruction in oncologic distal radius surgery [14,38,39].

## 5. Conclusions

Segmental resection of the distal radius and replacement with a proximal fibular autograft combined with DOB reconstruction for the management of Campanacci grade III giant cell tumors enables radical oncologic excision while preserving a satisfactory level of upper limb function, particularly in young and active patients for whom maintenance of forearm motion is essential for social and professional reintegration.

Integration of distal oblique bundle reconstruction contributes meaningfully to restoration of distal radioulnar joint stability without compromising oncologic radicality. The stability achieved through this ligamentous reconstruction supports preservation of pronation and supination, which are critical determinants of functional forearm performance and may reduce the risk of late distal radioulnar joint instability.

Compared with wrist arthrodesis, autologous reconstruction using a proximal fibular autograft offers the advantage of preserving wrist flexion and extension, functions that are irreversibly lost following definitive fusion. In addition, preservation of the articular cartilage may provide a biological benefit by reducing susceptibility to local tumor recurrence and maintaining reconstructive options should further surgical intervention become necessary.

Temporary stabilization using Kirschner wires, combined with structured immobilization and rehabilitation, allowed effective management of early postoperative instability without adversely affecting graft integration or functional recovery. The early postoperative radiocarpal instability observed in this case likely reflects the intrinsic biomechanical vulnerability of osteoarticular fibular reconstruction, particularly in the context of extensive capsular resection and subtle graft length sensitivity. Even minimal discrepancies in graft length or soft tissue tension may alter radiocarpal load distribution during the early healing phase. This observation underscores the importance of precise restoration of radial length, careful soft tissue balancing, and early radiographic surveillance. The need for temporary carpal stabilization represented a reactive measure in response to early instability rather than a mandatory component of the integrated technique; however, prophylactic temporary stabilization may be considered in selected high-risk cases.

The presented data are derived from a single illustrative case, which limits the ability to generalize clinical outcomes. Longer follow-up and larger case series will be necessary to validate durability and reproducibility of this technique. Follow-up duration remains limited, and longer-term evaluation is necessary to assess durability of functional outcomes and the occurrence of late complications, including potential degenerative changes at the radiocarpal or distal radioulnar joint. Should progressive arthritic degeneration develop over time, secondary procedures such as wrist arthrodesis may become necessary as part of the reconstructive spectrum. Functional assessment relied on the Musculoskeletal Tumor Society score, which, although validated for oncologic reconstruction, does not fully capture wrist-specific function or subtle deficits in forearm rotation. These factors should be considered when interpreting the reported outcomes.

The available clinical findings from this illustrative case support the technical feasibility of combining proximal fibular autograft reconstruction with distal oblique bundle stabilization. Broader validation will require application in larger clinical series. In the present illustrative case, tumor control was achieved and wrist arthrodesis was not required during the available follow-up period. These findings support the technical feasibility of combining radical resection with preservation of distal forearm function.

## Figures and Tables

**Table 1:** Campanacci classification of giant cell tumors.

Campanacci Grade	Characteristics
Grade I	Intracompartmental lesions, well delineated, with sclerotic margins and an intact outer cortex, without significant expansion.
Grade II	Active lesions, with relatively clear margins and no evident perilesional sclerosis, associated with cortical thinning and bone deformity, without invasion of the surrounding soft tissues.
Grade III	Aggressive tumors, with extracortical invasion into soft tissues, indistinct margins, and marked compromise of bone architecture.

**Table 2:** Postoperative Musculoskeletal Tumor Society (MSTS) functional scores over time.

Parameter	Immediate Postoperative	1 Month	3 Months	9 Months
Pain	1	1	2	5
Function	1	1	1	3
Emotional acceptance	2	3	3	5
Hand positioning	2	2	2	4
Manual dexterity	2	2	2	4
Lifting ability	0	0	0	3
Total (Percentage)	8 (26.7%)	9 (30.0%)	10 (33.3%)	24 (80%)

**Table 3:** Wrist range of motion at 9-month follow-up.

Motion	Range (Degrees)	Normal ROM (Degrees)	Percentage of Normal (%)
Wrist flexion	30	80	37.5
Wrist extension	45	70	64.3
Pronation	55	80	68.8
Supination	65	80	81.3

## Data Availability

The original contributions presented in this study are included in the article. Further inquiries can be directed to the corresponding author.

## Abbreviations

The following abbreviations are used in this manuscript:

GCT	Giant Cell Tumor
DRUJ	Distal Radioulnar Joint
DOB	Distal Oblique Bundle
MSTS	Musculoskeletal Tumor Society
